# Holdfast spreading and thickening during *Caulobacter crescentus* attachment to surfaces

**DOI:** 10.1186/1471-2180-13-139

**Published:** 2013-06-19

**Authors:** Guanglai Li, Yves V Brun, Jay X Tang

**Affiliations:** 1 Physics Department, Brown University, Providence, RI 02912, USA; 2 Institute of Molecular and Nanoscale Innovation, Brown University, Providence, RI 02912, USA; 3 Department of Biology, Indiana University, Bloomington, IN 47405, USA

**Keywords:** Caulobacter, Bioadhesives, Bacterial adhesion, Differentiation, Biofouling, Biofilms

## Abstract

**Background:**

Adhesion to surfaces facilitates many crucial functions of microbes in their natural habitats. Thus understanding the mechanism of microbial adhesion is of broad interest to the microbiology research community.

**Results:**

We report a study by fluorescence imaging and atomic force microscopy on the growth in size and thickness of the holdfast of synchronized *Caulobacter crescentus* cells as they attach to a glass surface. We found that the holdfast undergoes a two-stage process of spreading and thickening during its morphogenesis. The holdfast first forms a thin plate on the surface. The diameter of the holdfast plate reaches its final average value of 360 nm by the cell age of ~ 30 min, while its thickness further increases until the age of ~ 60 min. Our AFM analysis indicates that the holdfast is typically thicker in the middle, with gradual falloff in thickness towards the outer edge.

**Conclusions:**

We propose that the newly secreted holdfast substance is fluid-like. It has strong affinity to the surface and cures to form a plate-like holdfast capable of supporting strong and permanent adhesion.

## Background

In the environment, bacteria are predominantly attached to biotic or abiotic surfaces, where they are held by adhesive molecules at the surface of the cell envelope. Despite identification of adhesins in many bacterial species, little is known about the nature of the adhesive process from the material science point of view. In order to gain insight about the material properties of bacterial adhesins, we study the morphogenesis of the adhesive holdfast of the Gram negative bacterium *Caulobacter crescentus*. *C. crescentus* is a ubiquitous bacterium that can be found in wet soil and aquatic environments [1,2]. Its asymmetric cell division produces a motile swarmer cell and a sessile stalked cell. The swarmer cell swims by rotating its single polar flagellum [3-6]. This mechanism allows for dispersal of the progeny cells following each division, which reduces local competition for nutrients. The swarmer cell also harbors pili, which are synthesized at the flagellar pole immediately after cell division [7]. The stalked cell is typically attached to a surface by a holdfast found at the end of a thin, elongated extension of the cell envelope, called a stalk. The stalk is thought to increase nutrient uptake, which is particularly important in nutrient-deficient environments where molecular uptake is limited by diffusion [8].

The flagellum, pili, and the holdfast play important roles in surface adhesion [9-11]. Reversible adhesion occurs in swarmer cells where initial surface interactions are mediated by the flagellum and pili [12]. Contact of the flagellum and pili with a surface increases the load on the flagellum motor, halting flagellum rotation and triggering just-in-time deployment of holdfast from the flagellar pole. The attached cell subsequently develops into a stalked cell with elongation of a thin stalk from the pole bearing the holdfast. In cells that do not contact a surface, holdfast synthesis is regulated by the developmental program and occurs in the late swarmer stage [11,12]. There has not been much study with respect to possible differences between these two pathways, since the contact-triggered *C. crescentus* adhesion pathway has only been discovered recently [12].

The *C. crescentus* holdfast is a complex of polysaccharides and proteins required for adhesion to surfaces with impressive strength [9,13-15]. The fluorescently labeled lectin fluorescein isothiocyanate-wheat germ agglutinin (FITC-WGA), which binds to oligomers of N-acetylglucosamine (GlcNac or NAG), binds specifically to the holdfast, indicating that the holdfast contains NAG [13]. Furthermore, the holdfast is sensitive to treatment with lysozyme, which cleaves NAG polymers [13,16]. Mutants that cannot be stained with FITC-WGA are unable to form irreversible surface adhesion [13].

In this paper, we used fluorescence microscopy and atomic force microscopy to study the holdfast growth of cells attached to a surface. We show that the holdfast undergoes a two-stage process of spreading and thickening during its morphogenesis. Based on the observed holdfast growth characteristics, we propose that the newly secreted holdfast material is a fluid-like substance that cures to form a plate-like holdfast capable of supporting strong and permanent adhesion.

## Methods

### Strain and synchronization

Wild-type *C. crescentus* strain CB15 was cultured in a peptone-yeast extract (PYE) medium [1] at 30°C. Synchronized swarmer cells were obtained using a plate releasing technique [12,17]. Unless specified, the synchronized cells were harvested 5 min or less after cell division. The age variance of these cells, with time counted from separation and release of the swarmer cell, was within 5 min. In selected experiments, young swarmer cells were also synchronized to a narrower range of within 1 min in age in order to best resolve the early stages of holdfast development.

### Fluorescence labeling of holdfasts

Holdfasts were labeled as described previously [12]. A drop of synchronized swarmer cells was placed on a coverslip for 5 min, allowing some swarmer cells to attach to the glass surface. For the study of cells younger than 6.5 min, incubation time was reduced to 1 min. The unattached cells were rinsed off gently with fresh PYE and the cells attached to the coverslip were then grown at 30°C for various lengths of time. After growth, the coverslip was rinsed with water to remove nutrients. Cells were labeled with fluorescein-conjugated WGA solution on ice for various amounts of time, supplemented with 0.05% (w/v) sodium azide to stop cell growth during the labeling. The concentration of the fluorescein-WGA varied from 0.02 to 1 mg/ml. After labeling, the coverslip was rinsed with the sodium azide solution three times and an anti-photobleaching solution was added to the coverslip prior to fluorescence microscopy. The anti-bleaching solution contained 20 μg/ml catalase, 0.5 mg/ml glucose, 0.1 mg/ml glucose oxidase, and 0.25 vol% ß-mercaptoethanol [18].

### Fluorescence microscopy

A Nikon Eclipse E800 epifluorescence microscope with a 100 × oil immersion objective lens (Plan Apo) was used to image the fluorescently labeled holdfast. A highly sensitive and linear CoolSnap camera was used to record the fluorescence images of holdfasts, controlled by MetaMorph (Universal Imaging, PA) software. The attached cells were first brought into focus under phase contrast setting for easy location of the cells. Then the holdfasts were observed under fluorescence mode with fine adjustment of focus. Consecutive fluorescence images were taken with 0.1 s exposure time while manually adjusting the focus with the fine adjustment knob. Optimal focus was achieved within ten attempts. The image of the 10th exposure was used to obtain the fluorescence intensities of holdfasts.

### Measurement of fluorescence intensity

To measure the integrated fluorescence intensity, a circle larger than the holdfast image was drawn using the imaging software and the intensity was integrated over all the pixels inside the circle. The sum was then subtracted by the integrated background intensity of a nearby circle of the same size to obtain the integrated intensity of the holdfast. This method eliminates background intensity from the camera noise and from dye molecules adsorbed on the glass surface. The net integrated fluorescence intensity of holdfasts was measured for over 500 cells older than 7.5 min in age per time point. The fluorescence images of most holdfasts were sufficiently bright and their intensities were measured by an automated routine using the commercial software Matlab (Mathworks, Natick, MA, USA). A small sub-population of holdfasts were too dim to be recognized by the Matlab program and their intensities were determined individually by the integrated intensity function in MetaMorph. For cells younger than 6.5 min, fluorescence intensities of almost all holdfasts were too weak to be recognized by the Matlab program. Instead, about 100 holdfasts at each chosen age were measured individually using MetaMorph.

### Selection of experimental condition for quantitative fluorescence analysis

We used the following method to determine proper fluorescein-WGA labeling conditions. Synchronized swarmer cells were allowed to quickly attach to a glass microscope coverslip. The unattached cells were washed away. The attached cells were incubated for 27.5 min at 30°C to ensure formation of holdfasts. We then measured average intensity of those holdfasts labeled with 20, 100, and 500 μg/ml fluorescein-WGA for 15 min and average intensity of holdfasts labeled with 100 μg/ml fluorescein-WGA for 5, 10, 15 and 20 min in order to determine the dependence of the average integrated fluorescence intensity on dye concentration and incubation time. We found that the integrated fluorescence intensity was not sensitive to the lectin concentration or labeling time within these ranges, suggesting saturation of dye labeling under these experimental conditions. We concluded that performing labeling experiments within these ranges allows robust comparison of holdfast size even with somewhat variable amounts of dye and incubation time, as long as they were above 20 μg/ml and 5 min, respectively. For all subsequent experiments, we labeled the holdfasts with 100 μg/ml lectin for 15 min.

### Atomic force microscopy (AFM)

In order to obtain a clean surface as a substrate for AFM imaging, glass coverslips were soaked in a solution of 6 % (w/v) Nochromix (GODAX Laboratories, Inc.) in concentrated H_2_SO_4_ for 1 hour and then rinsed thoroughly with deionized water. A drop of culture containing synchronized swarmer cells was placed on a clean coverslip for 5 min. The unattached cells were rinsed off with oxygenated fresh PYE and the attached cells were then grown at 30 °C over various time intervals to allow for holdfast growth. The coverslip was then blow-dried gently with compressed N_2_ gas so that the attached cells fell over to the side, getting stuck and dried onto the glass surface. The dried cells and their holdfasts, also dried on the glass surface, were imaged using a Nanoscope IIIa Dimension 3100 (Digital Instruments, Santa Barbara, CA) atomic force microscope using contact mode in air.

## Results

### Distribution of holdfast fluorescence intensity at various ages

**Figure 1:**
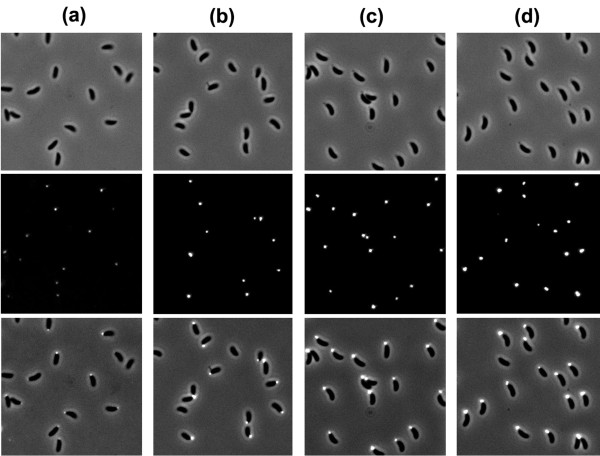
**Holdfast secretion level at different ages, detected by labeling with 100 μg/ml fluorescein-WGA-lectin for 15 min on ice, (a) 7.5 ± 2.5 min, (b) 17.5 ± 2.5 min, (c) 27.5 ± 2.5 min, and (d) 37.5 ± 2.5 min.** Top panel shows phase contrast images, middle panel fluorescence images, and bottom panel the combined phase and fluorescence images.

**Figure 2:**
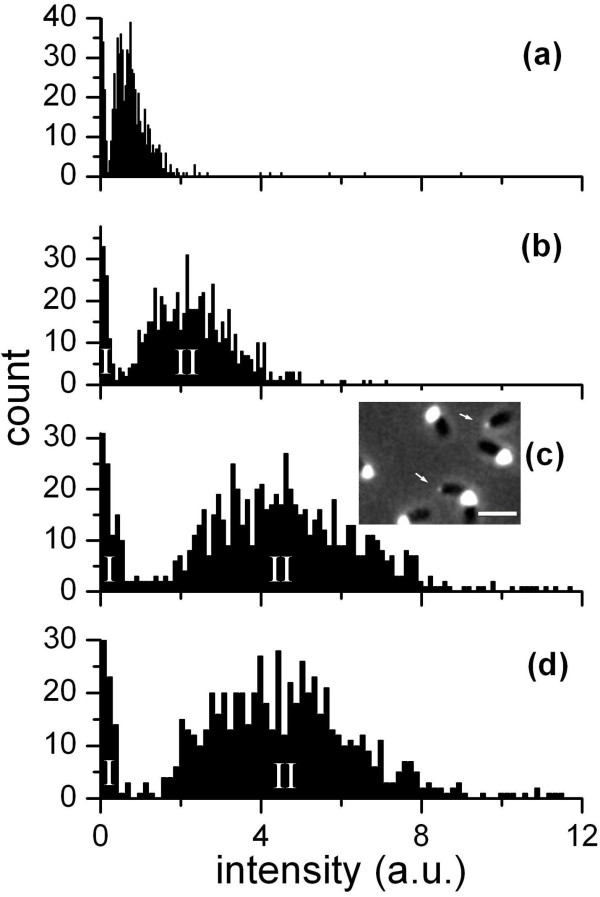
**Fluorescence intensity distribution of holdfast at various ages labeled with 100 μg/ml fluorescein-WGA-lectin, (a) 7.5 ± 2.5 min, (b) 17.5 ± 2.5 min, (c) 27.5 ± 2.5 min, and (d) 37.5 ± 2.5 min.** Note that the intensities fall into two groups, indicated as I and II. Approximately 10% of holdfasts are in group I, whose intensities remain very low. Inset in (**c**) is a combined phase and fluorescence image of 27.5 ± 2.5 min old cells, showing a few examples of the two groups of holdfasts with different fluorescence intensities. The fluorescence intensities of two holdfasts indicated by arrows are much weaker than the others. These two cells are identified as group I cells in co-existence with several group II cells.

Fluorescein-WGA labeling confirmed the previous report that young swarmer cells start secreting holdfast within minutes following their attachment [12]. Figure 1 shows phase contrast and fluorescence images of cells at various ages. Holdfasts were clearly visible for attached cells as young as 7.5 min old. The intensity increased with age but the difference between holdfasts of 27.5 and 37.5 min old cells became insignificant. Analysis of the fluorescence intensity of labeled holdfast showed a wide variation in intensity at each time point (Figure 2). This result suggests that the holdfasts of different cells grow at different rates, and that the final sizes of the holdfast vary significantly from cell to cell. Interestingly, the intensities of the holdfasts fell into two groups, marked as I and II in Figure 2. Examples of each group of cells at age of 27.5 min are shown in the inset of Figure 2c. Holdfasts of group I have very weak intensities, less than one tenth of those in group II on average. Approximately 10% of holdfasts fell into group I. This intriguing result was reproducible among several experiments. Since the cells from each experiment came from clonal populations, it is unclear what causes the bimodal distribution in holdfast fluorescence intensity.

**Figure 3:**
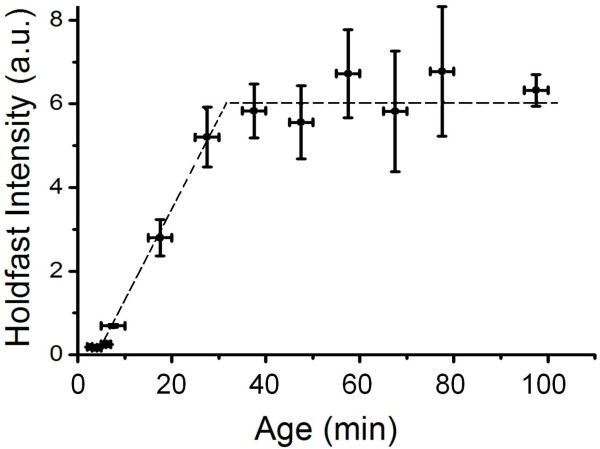
**Growth of holdfast attached to a solid surface measured with fluorescence microscopy.** This figure shows the fluorescence intensity of holdfast as a function of cell age. Each data point is the average over two or three samples. Error bars are the standard error. The dotted lines are drawn as a guide to the eye.

We found that the average fluorescence intensity of holdfasts increased with cell age during the first 30 min but then saturated at a constant level (Figure 3). Since the labeling step was done following different times of holdfast growth, our data suggest either that the attached cells stopped secreting more holdfast after about 30 min, or that the holdfast continued to thicken after 30 min, but if the fluorescein-WGA only bound to the surface of the dense holdfast material the fluorescence intensity would no longer increase noticeably as the holdfast layer continued to thicken. We turned to AFM analysis below in order to distinguish between these possibilities.

### The holdfast spreads to a thin plate at the attachment site

**Figure 4:**
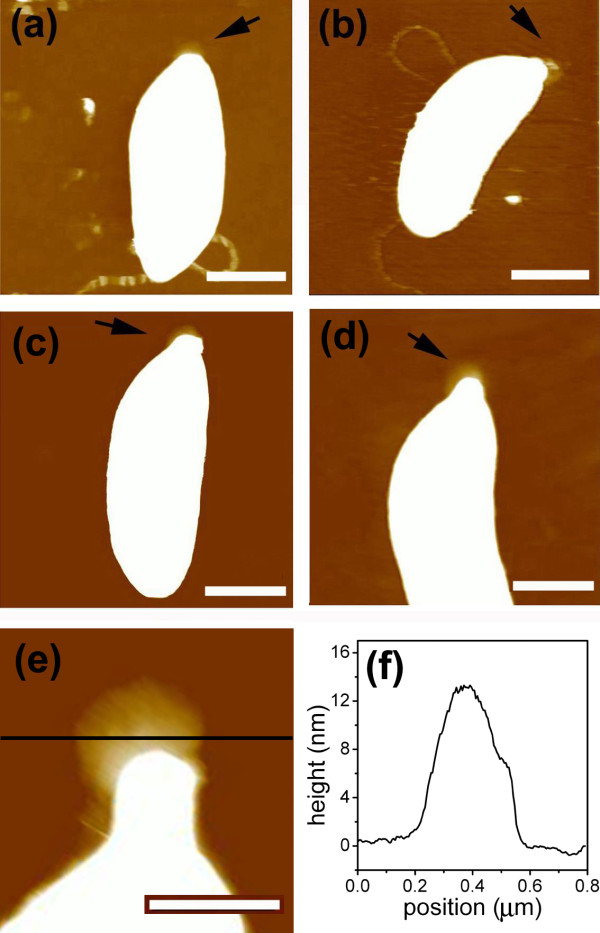
**AFM images of dried holdfasts of cells at various ages, (a) 17.5 ± 2.5 min, (b) 27.5 ± 2.5 min, (c) 37.5 ± 2.5 min, (d) 47.5 ± 2.5 min, and (e) 57.5 ± 2.5 min.** Scale bars represent 400 nm. A black line is drawn through the center of the holdfast. (**f**) is the height profile along the black line in (**e**), showing both the height and width of the holdfast.

Previous studies have used electron microscopy or FITC-WGA labeling to measure holdfasts [13,14]. While these methods provided useful information about holdfast size, AFM can be used to measure holdfast size in three-dimensions [9,16]. In order to directly analyze holdfast synthesis by AFM, swarmer cells were synchronized by the plate release method. They were allowed to quickly attach to a glass microscope coverslip. After the unattached cells were washed away, attached cells were allowed to grow for different amounts of time before drying and imaging by AFM. Figure 4 shows typical AFM images of cells at different ages. The cell body laid down on the surface during the drying procedure and typically only a part of the holdfast was approachable by the AFM tip. In very young cells, the cell body occluded the holdfast. For instance, AFM could not image the holdfast of 7.5 min old cells. The holdfasts of 17.5 and 27.5 min old cells were larger and partially detectable. For cells over 37.5 min old, a thin stalk appeared, so most of the holdfast area became detectable at the tip of the stalk. The edge of the holdfast was clearly discernible in Figure 4e, and was roughly circular. The holdfast became gradually thinner towards the edge, taking the shape of a suction cup. Figure 4f is the height profile along a dark line drawn visually through the center of the holdfast in Figure 4e, showing that this holdfast is ~ 400 nm in diameter, 12 nm thick in the center, and thinner at the edge. We previously showed that holdfasts have the properties of a polysaccharide gel, with wet holdfasts approximately 4 times as thick as when they were dried [16]. With this correction factor, the thickness of wet holdfasts would be between 40 and 50 nm, which is still only about one tenth of their diameter. We conclude that the holdfast of *C. crescentus* has the structure of a thin plate.

### The holdfast undergoes a two-stage process of spreading and thickening

**Figure 5:**
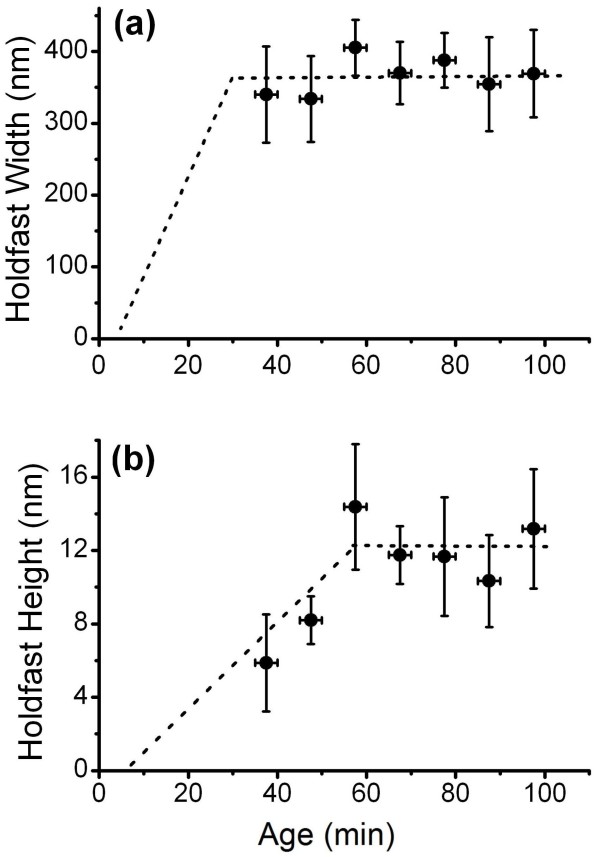
**Growth of holdfast attached to a surface measured with AFM.** (**a**) and (**b**) are the diameter and thickness of dried holdfast measured from AFM images as functions of cell age, averaged over 20 holdfasts for each data point. The error bars are standard errors. The dashed lines are drawn as guide to the eye.

Further AFM measurements were conducted to probe the dynamics of holdfast morphogenesis. Figure 5 shows holdfast diameter and thickness as measured by AFM. The holdfast diameter was quite stable and averaged ~ 360 nm for cells older than 37.5 min, indicating that the holdfast had already attained its maximum diameter at 37.5 min (Figure 5a). We could not reliably measure the holdfast of cells younger than 37.5 min old by AFM because they tended to be blocked by the cell body. This result is consistent with the fluorescence data, showing no further increase in intensity beyond the cell age of 35 min (Figure 3). In contrast, holdfast thickness continued to thicken over the next 30 min to about 12 nm in 57.5 min old cells (Figure 5b). The lack of corresponding increase in fluorescence labeling suggests that fluorescein-WGA predominantly labels the surface of the holdfast, which would remain essentially constant as the thin holdfast gradually thickened. Growth in holdfast thickness stopped approximately by the time the attached cells entered their pre-divisional stage. Our experiment did not extend beyond the first cell cycle, thus it is unclear whether holdfast secretion resumes during subsequent cycles of division.

## Discussion

**Figure 6:**
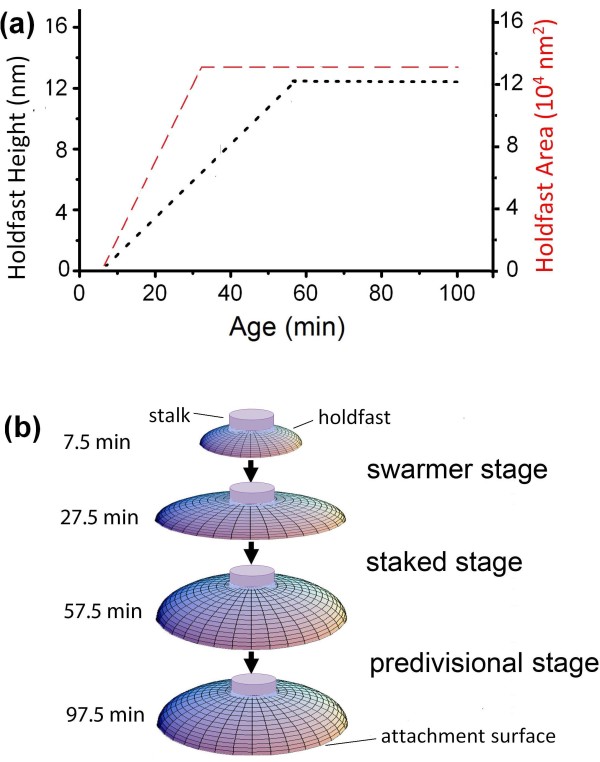
**Illustration of growth in size and shape of holdfast following a *C. crescentus *cell’s attachment to a solid surface.** (**a**) A recap of holdfast growth based on fluorescence (area) and AFM (area and height) measurements. (**b**) Schematics illustrating the spread, thickening, and stabilization of a holdfast as the cell that produces it goes through developmental stages.

The above results suggest how an attached *C. crescentus* cell develops its holdfast over time, depicted illustratively in Figure 6. Shortly after attachment, the cell starts to secrete holdfast polysaccharide. This material spreads rapidly on the submerged surface to form a thin plate. With more holdfast material secreted over time, the width of the plate increases with age to a final size at about 360 nm shortly after the cell enters the stalked stage. The limited holdfast width suggests that the adhesive material likely cures upon contact with the surface to quickly provide an effective adhesion after secretion. Then the spreading stops, but the holdfast continues to thicken. The simplest interpretation is that more holdfast polysaccharide continues to be secreted. Newly secreted material increases the thickness of the plate until the cell age of 57.5 min. The final shape of the holdfast is thin at the edge and thicker in the middle, presumably optimized for good adhesion strength. Indeed, we have previously showed that a fully cured holdfast yields adhesion forces in the micro-newton range [9], which is to our knowledge the strongest among natural glues.

The distinct time course for the spreading and thickening of a new holdfast offers important insights into the material properties of the holdfast. Newly secreted holdfast material appears to behave as a viscous fluid, which spreads quickly over a flat solid surface. The physics phenomenon is akin to what is often called “wetting” [19,20], typically a process during which a liquid drop spreads over a solid surface in the ambient environment. For this analogy to be valid the holdfast material must not mix with the growth medium and there ought be significant surface tension at the holdfast/medium interface. In addition, the holdfast must have strong affinity for the surface. All these conditions appear to have been met, leading to the adhesion characteristics observed.

The AFM images and particularly the height scan as illustrated in Figure 5b offer further insights on the curing process of newly secreted holdfast material. Because holdfasts are thin and the contact angle at the edge of the holdfast is small, the size of the holdfast does not appear to be caused by balancing the forces of line tension at the contact edge and the weight of the spreading liquid drop. Instead, the holdfast size may be dictated by the rate of gelation of the holdfast. Once the first thin layer is cured, the additional secretion might spread over the gelled disk and cures in comparable or even shorter amounts of time, thus continually thickening the gelled holdfast until the secretion stops. The fact that the holdfast stops spreading but continues to thicken indicates that some kind of molecular transformation takes place faster than the time for the new secretion to spread past the footprint of the holdfast cured from the initial spread. *Caulobacter* cells can adhere strongly to a wide variety of surfaces, including glass, plastics, and metals [10,13]. The non-specific nature of these strong interactions implies that they are non-covalent and most likely attributable to van der Waals forces [21,22]. The major component of the holdfast, polymers of *N*-acetylglucosamine, may be well suited as the base material for a wet adhesive. It appears to produce strong molecular interactions with many solid materials due to non-specific interactions; it does not disperse in an aqueous environment upon secretion due to a high degree of crosslinking. Unfortunately, the detailed composition of the holdfast remains unknown and we know nothing about the processes that triggers the curing of newly secreted holdfast material.

## Conclusions

Adhesives have a broad range of biomedical applications, from denture to surgical suture. A good bio-adhesive must be fast to cure, waterproof, and resilient once bonded with a range of different materials. A synthetic adhesive often relies on catalytic reactions to cure, such as in an epoxy-resin mixture. The curing of adhesive mixtures for medical and dental applications is typically triggered by UV light, which conveniently triggers crosslinking reactions at the desirable site. Most natural biological adhesins, such as the holdfasts secreted by *Caulobacter crescentus* and several species of alphaproteobacteria [23-25], adhere to solid surfaces under normal aqueous conditions. This important property naturally selected during the course of evolution may soon be harnessed for biomedical applications.

## Competing interests

The authors declare that they have no competing interests.

## Authors’ contributions

GL participated in the project design, performed the experiments, and drafted the manuscript. YVB participated in the project design and coordination. He also contributed to the manuscript revision. JXT conceived of the study, led the project design, coordination and manuscript revision. All authors read and approved the final manuscript.
